# *Salmonella enterica* and outer membrane vesicles are current and future options for cancer treatment

**DOI:** 10.3389/fcimb.2023.1293351

**Published:** 2023-12-05

**Authors:** Genesy Pérez Jorge, Marco Túlio Pardini Gontijo, Marcelo Brocchi

**Affiliations:** ^1^ Universidade Estadual de Campinas (UNICAMP), Departamento de Genética, Evolução, Microbiologia e Imunologia, Laboratório de Doenças Tropicais, Instituto de Biologia, Campinas, Brazil; ^2^ Department of Molecular Genetics and Microbiology, Duke University School of Medicine, Durham, NC, United States

**Keywords:** delivery vector, combination therapy, nanocarriers, outer membrane vesicles, *Salmonella* Typhimurium, immunotherapy, cancer, anticancer

## Abstract

Conventional cancer therapies have many limitations. In the last decade, it has been suggested that bacteria-mediated immunotherapy may circumvent the restrictions of traditional treatments. For example, *Salmonella enterica* is the most promising bacteria for treating cancer due to its intrinsic abilities, such as killing tumor cells, targeting, penetrating, and proliferating into the tumor. *S. enterica* has been genetically modified to ensure safety and increase its intrinsic antitumor efficacy. This bacterium has been used as a vector for delivering anticancer agents and as a combination therapy with chemotherapy, radiotherapy, or photothermic. Recent studies have reported the antitumor efficacy of outer membrane vesicles (OMVs) derived from *S. enterica*. OMVs are considered safer than attenuated bacteria and can stimulate the immune system as they comprise most of the immunogens found on the surface of their parent bacteria. Furthermore, OMVs can also be used as nanocarriers for antitumor agents. This review describes the advances in *S. enterica* as immunotherapy against cancer and the mechanisms by which *Salmonella* fights cancer. We also highlight the use of OMVs as immunotherapy and nanocarriers of anticancer agents. OMVs derived from *S. enterica* are innovative and promising strategies requiring further investigation.

## Introduction

Cancer is recognized as a major global burden on the public health system that lacks fully effective therapy (Sung et al., 2021). Conventional cancer therapies, such as surgical resection, radiotherapy, and chemotherapy, have many limitations, including the inability to fight metastatic tumors, high toxicity in healthy tissues and cells, the development of multiple adverse effects that can considerably reduce the quality of life of patients and the possible appearance of aggressive and chemotherapy-resistant phenotypes (Dillekås et al., 2016; Zhou et al., 2018). Additionally, the poor vasculature in tumors and the extracellular matrix around the tumor, also called desmoplasia, makes it difficult for chemotherapeutic drugs to penetrate (Binnewies et al., 2018; Ebelt et al., 2020). Unsatisfactory results and limitations of conventional cancer therapy led to the development of new alternative cancer treatments like immunotherapy.

**Figure 1:**
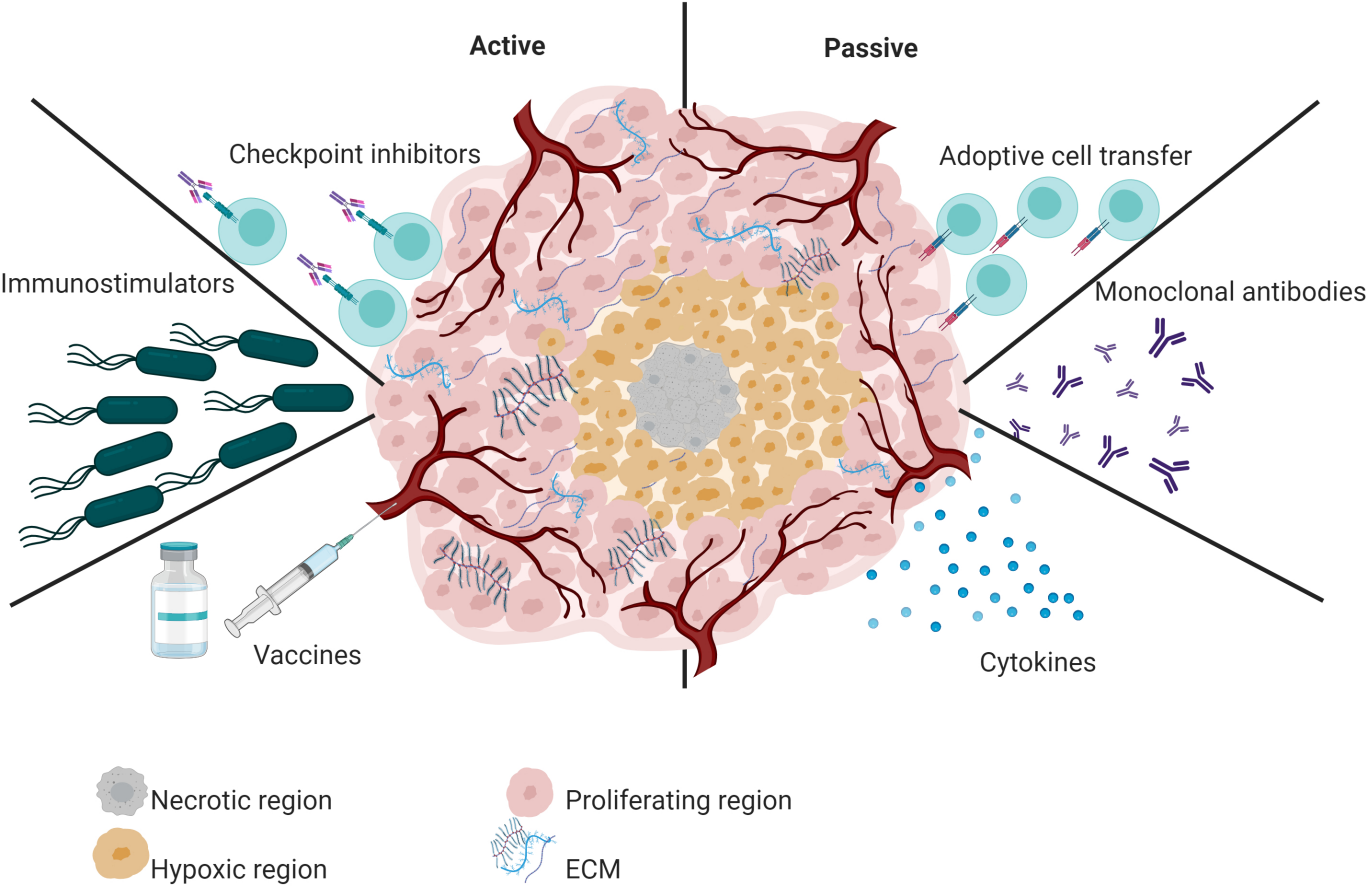
Overview of active and passive immunotherapy strategies. Active immunotherapy includes the administration of vaccines, checkpoint inhibitors, and immunostimulators such as attenuated bacteria and bacterial OMVs. In contrast, passive immunotherapy refers to tumor-specific monoclonal antibodies, cytokines, or adoptive T cells.

Immunotherapy has had a significant impact on oncology in the last decade (Gao et al., 2020), which has been evidenced by the Food and Drug Administration (FDA) approval of various immunotherapeutic strategies for use in humans (Galluzzi et al., 2014). Based on the ability to reactivate the host’s immune system against cancer cells and the mechanism of action of the immunotherapeutic agent used, immunotherapy strategies can be classified as passive or active (Auslander et al., 2018; Galon and Bruni, 2019) (**Figure 1**).

Passive immunotherapy does not exploit the immune system’s natural response. Instead, it delivers immune system components, such as targeted monoclonal antibodies (O’Brien et al., 2022) or adoptive T cells (Tran et al., 2014). For example, the FDA-approved monoclonal antibody pembrolizumab significantly improved the survival of advanced non-small-cell lung cancer patients compared to placebo (O’Brien et al., 2022). Similarly, tebentafusp-tebn improved survival in patients with metastatic uveal melanoma (Dhillon, 2022). Another FDA-approved monoclonal antibody is Nivolumab. This immunotherapeutic treatment is used to treat multiple types of cancer in advanced stages (Paik, 2022). Concerning T cell-based immunotherapy, the FDA has approved six CAR T-cell therapies (Tisagenlecleucel, axicabtagene ciloleucel, brexucabtagene autoleucel, Lisocabtagene maraleucel, Idecabtagene vicleucel and Ciltacabtagene autoleucel). This type of treatment has shown excellent efficacy against hematological malignancies (Khan et al., 2022).

In contrast, in active immunotherapy, the host’s immune system plays a major role; Active immunotherapy induces a durable memory response. The most relevant active immunotherapy strategies include cancer vaccines (Pol et al., 2015) and immunostimulatory, such as attenuated bacteria and their derivatives (outer membrane vesicles-OMVs) (Liang et al., 2021). FDA-approved cancer vaccines vary by platform. One of the oldest and most used is TheraCys. This vaccine was designed using live Bacillus Calmette–Guérin bacteria (live intravesical BCG). BCG is used to treat urothelial carcinoma *in situ* of the bladder after transurethral resection. Although BCG treatment has increased the survival of patients with bladder cancer, a subset of patients does not respond to BCG treatment (Chen et al., 2023a). On the other hand, the FDA has also approved immunotherapies based on viral platforms. IMLYGIC or T-VEC is an oncolytic viral therapy (based on herpes simplex virus 1) for treating advanced melanoma. Treatment with IMLYGIC statistically improved complete response or partial response rates in patients with melanoma. However, this therapy has limitations, such as cold chain requirements and intralesional administration (Shalhout et al., 2023).

Although bacteria are disease-causing microorganisms, different studies have shown that bacteria have the potential for cancer therapy (Yang et al., 2023). Even bacterial-mediated immunotherapy against cancer is one of the most promising strategies (Tran et al., 2014; Galon and Bruni, 2019; Mi et al., 2019). Bacterial-mediated immunotherapy may circumvent the limitations of conventional treatments; different genera of bacteria, such as *Salmonella* (Felgner et al., 2020), *Escherichia* (Kang et al., 2020)*, Clostridium* (Staedtke et al., 2016), and *Listeria* (Vitiello et al., 2019) have been the focus of research and have shown antitumor activity. Among them, *Salmonella enterica* subsp. *enterica* serovar Typhimurium (*S.* Typhimurium) stands out considerably as the most studied bacteria for cancer treatment (Zhou et al., 2018).

*S enterica* has been extensively evaluated in preclinical cancer studies, and its antitumor potential has been demonstrated in both solid and metastatic tumors (Toso et al., 2002; Mi et al., 2019). In this review, we summarize recent advances in *S. enterica*-mediated cancer immunotherapy and the mechanisms by which *S.* e*nterica* fights cancer. For a broader perspective on OMVs-based vaccines, please refer to Gao et al. (2022) and Jalalifar et al. (2023).

In addition, we also describe the promising new use of *Salmonella*-derived OMVs in cancer therapy. To begin with, we will introduce the genomic advantages of *S. enterica* that enable its modification to convert pathogenic strains to safe strains for use in humans or to synthesize antitumor agents (Galen et al., 2016). In addition, we highlight the intrinsic ability of *S. enterica* to precisely target tumors by detecting chemical signals found in the tumor microenvironment (Kasinskas and Forbes, 2007).

Furthermore, we also point out that *S. enterica* prefers to selectively accumulate and proliferate in the tumor microenvironment, considerably reducing toxicity in normal tissues. Subsequently, we focus on the immunological mechanisms described by which *S. enterica* exerts its antitumor activity. We then describe the use of this bacterium as a vector of antitumor agents and its combination with conventional treatments. Finally, we highlight the use of OMVs derived from *S. enterica* in cancer treatment. For more information on the interaction mechanisms between OMVs and host cells, please refer to (Wang et al., 2022). *Salmonella*-derived OMVs in cancer treatment is a new and little-explored strategy. However, the currently available evidence suggests its use in cancer immunotherapy is promising.

## 2 Advantages of using *Salmonella enteric* over conventional therapies

### 2.1 Versatile genetic programmability

*S. enterica* genome can be genetically engineered like a programmable robot to ensure safety and increase its therapeutic activity. The construction of safe strains of *S. enterica* through the attenuation of virulence is a research focus. Virulent strains of *S. enterica* can be attenuated through the deletion or mutation of virulence (Frahm et al., 2015), metabolic (Miyake et al., 2019), or regulatory genes (Hirsch Werle et al., 2016). For example, to increase the safety of *S.* Typhimurium, the well-known strain VNP20009 was built through the chromosomal deletion of the *purI* and *msbB* genes. These modifications generated a strain with an LD_50_ increased by more than 10,000 times in mice (Clairmont et al., 2000). The *purI* gene is necessary for the synthesis of adenine; therefore, strains lacking *purI* require an exogenous source of purine. This genetic modification improves the ability of the bacteria to grow in tumor tissues rich in purine (Yam et al., 2010). Therefore, *S. enterica* strains lacking the *pur*I gene can have a more significant multiplication in tumors than in normal tissues.

The deletion of the *msbB* gene, for example, decreases septic shock (Low et al., 1999). The deletion of this gene causes the *S.* Typhimurium to induce a lower inflammatory response due to the lack of terminal myristoylation of lipid A of lipopolysaccharide (LPS), generating a strain with an excellent safety profile that retains the ability to accumulate in the tumor and suppress the tumor *in vivo* and *in vitro* (Low et al., 1999; Clairmont et al., 2000; Toso et al., 2002; Zhang et al., 2016). Genetic modifications in LPS can improve the safety profile of *S. enterica* (Frahm et al., 2015; Liang et al., 2022). It has been shown that strains with deletions in LPS are less likely to induce sepsis and are more susceptible to the immune system, making their application safer in cancer patients (Felgner et al., 2016b; Felgner et al., 2018). Different mutants were constructed by deleting genes involved in synthesizing *S.* Typhimurium LPS. Among these, the Δ*rfaG* mutant showed an excellent safety profile in mice and a high tumor targeting. However, its efficacy was compromised. To find the correct balance between attenuation and therapeutic efficacy, the Δ*rfaG* mutant was complemented with the *rfaG* gene under a promoter regulated by the sugar arabinose. This additional modification allows the Δ*rfaG* mutant to display wild-type LPS in the presence of arabinose (Frahm et al., 2015). However, the mutant exhibits incomplete LPS and an attenuated phenotype in the mammalian host, where arabinose is absent. This strategy increased the therapeutic efficacy of the Δ*rfaG* mutant in murine models of colon and kidney cancer (Frahm et al., 2015). These and other results indicate that although the modifications in LPS represent an attractive strategy for constructing strains with a good safety profile, the right balance is essential so that these modifications do not affect antitumor abilities.

*S. enterica* strains deficient in amino acid biosynthesis have also been designed to increase antitumor capabilities and safety profile*. S.* Typhimurium A1-R (dependent on leucine and arginine) selectively targets and replicates in tumor tissues over normal tissues, probably because it receives these amino acids from the tumor microenvironment (Miyake et al., 2019). In normal tissues, A1-R does not persist, and its toxicity is low (Liu et al., 2010). The antitumor ability of this strain has been evaluated in different mouse models of pancreatic (Yam et al., 2010; Kawaguchi et al., 2018b; Murakami et al., 2018), cervical (Miyake et al., 2019), prostate (Zhao et al., 2005), lung (Liu et al., 2010), breast (Miwa et al., 2014), and melanoma (Kawaguchi et al., 2018b) and osteosarcoma (Hayashi et al., 2009), in doses ranging from 10^7^-10^9^ CFU, without severe toxicity. Other strains deficient in amino acids that have shown a good safety profile and antitumor effects are strains deficient in aromatic amino acid biosynthesis, such as the *aroA, aroC*, and *aroD* mutants (Grille et al., 2014; Jin et al., 2017; Yoon et al., 2017; Bascuas et al., 2018). In addition, *aro* mutants are attenuated when inoculated by different routes of administration (Yang et al., 2008; Guo et al., 2011; Bascuas et al., 2018). The attenuation of these mutants is due to their inability to replicate and survive in mammalian cells, which considerably improves their safety profile to the host.

Attenuating *S. enterica* virulence can be achieved by targeting genes that regulate gene expression (Phan et al., 2015; Hirsch Werle et al., 2016). Guanosine 5′-diphosphate-3′-diphosphate (ppGpp) is an essential regulator of *S. enterica* gene expression (Pizarro-Cerdá and Tedin, 2004). The avirulent *S.* Typhimurium ΔppGpp strain, unable to synthesize ppGpp, can suppress the growth of colon tumors (Phan et al., 2015; Xu et al., 2022). The host integration factor (*ihf*) can also regulate the expression of different *S*. Typhimurium genes, including virulence and pathogenicity genes (Hirsch Werle et al., 2016). Deleting the *ihfA* gene reduced the virulence of *S.* Typhimurium in mice without affecting its ability to invade and survive in mammalian cells. Our research group demonstrated that *S.* Typhimurium *ΔihfA* could inhibit the growth of melanoma tumors in a murine model (Hirsch Werle et al., 2016). Inactivation of genes that regulate gene expression is an attractive strategy to achieve attenuation of the virulence of *S. enterica.*

Several other attenuated strains of *S. enterica* have been designed and evaluated in cancer-mediated therapy (**Table 1**). Recently, *S*. Typhimurium KST0650 was hyperattenuated by induction of mutations by gamma irradiation and exhibited enhanced invasiveness and survival in tumor cells (Gao et al., 2020). *S*. Typhimurium YB1, derived from an *aroA* mutant (SL7207), was constructed by replacing the essential *asd* (aspartate-semialdehyde dehydrogenase) with a construct in which *asd* is under the control of a hypoxia-conditioned promoter. This strain selectively accumulated in the tumor (tumor-liver ratios of 3900:1), and colonization was detected in hypoxic and necrotic regions with little or no blood vessels (Yu et al., 2012). Although various genetic modifications have allowed the attenuation of different strains of *S.* Typhimurium to reduce virulence in healthy tissues, their therapeutic activity was compromised without additional modifications.

**Table 1:** Attenuated *Salmonella* Typhimurium strains used for cancer therapy in animal models.

Strain	Gene mutation	Antitumor agent delivered	Combined with	Tumor-bearing mice	Route (Dose (CFU))	Ref.
VNP20009	*ΔmsbBΔpurI*	HSV-TK		melanoma	iv (4x10^6^)	(Pawelek et al., 1997)
		shABCB5	Cyclophosphamide	Melanoma/colon carcinoma	ip (1x10^5^)	(Zhang et al., 2016)
		shIDO		colon adenocarcinoma	ro (5x10^6^)	(Phan et al., 2020)
			Photothermal therapy	melanoma	iv (1x10^5^)	(Chen et al., 2018a)
			Hydroxychloroquine liposomes	melanoma	iv (1x10^5^)	(Wang et al., 2018)
			Triptolide	melanoma	ip/iv (5x10^5^)	(Chen et al., 2017)
		shSox2	HM-3 angiogenesis inhibitor polypeptide	lung carcinoma	iv (2x10^6^)	(Zhao et al., 2016)
			N-FADD	melanoma	it (2x10^5^)	(Yang et al., 2016)
		CytosineDeaminase	5-FC	tumor tissue	iv (1x10^6^)	(King et al., 2002)
		IL-18		Colon carcinoma/breast carcinoma	iv (5x10^6^)	(Loeffler et al., 2008)
		anti-CEA scFv		adenocarcinoma	iv (2x10^6^)	(Bereta et al., 2007)
				ductal adenocarcinoma/leukemia	it (2x10^6^)	(Zhou et al., 2016; Li et al., 2020b)
		Methioninase		Prostate, hepatocellular, triple-negative breast cancer	iv (2x10^5^)	(Zhou et al., 2023)
VNP20009asd-	*ΔmsbBΔpurIΔasd*	carboxypeptidase G2	Prodrugs	Breast carcinoma/colon carcinoma/melanoma	iv (2x10^6^)	(Friedlos et al., 2008)
A1-R	leucine and arginine auxotrophic strain		Recombinant Methioninase	melanoma	iv (5x10^7^)	(Kawaguchi et al., 2018a)
			Gemcitabine	pancreaticcancer	Iv (5x10^7^)	(Kawaguchi et al., 2018b)
			nab-paclitaxel	Cervical cancer	iv (5x10^7^)	(Miyake et al., 2019)
			Methioninase and cisplatinum	osteosarcoma lung	iv (5x10^7^)	(Igarashi et al., 2018)
				pancreatic adenocarcinoma	iv (1x10^7^)	(Murakami et al., 2018)
				prostate adenocarcinoma	iv/it (2x10^9^)	(Zhao et al., 2005)
				lung carcinoma	iv (5x10^7^)	(Liu et al., 2010)
				osteosarcoma	iv (3x10^8^)	(Hayashi et al., 2009)
				Pancreatic adenocarcinoma	iv (5x10^7^)	(Yam et al., 2010)
				metastatic breast cancer	iv (5x10^7^)	(Miwa et al., 2014)
				breast tumor	iv (5x10^7^)	(Zhao et al., 2006)
ΔppGpp	*ΔrelA ΔSpoT*		Recombinant IL-1β or TNF-α	colon carcinoma	iv (4x10^7^)	(Kim et al., 2015)
		RGD peptide		Breast adenocarcinoma/melanoma/pancreatic adenocarcinoma/colon carcinoma	iv (3x10^7^)	(Park et al., 2016)
				colon adenocarcinoma	iv (4x10^7^)	(Phan et al., 2015)
		Flagellin B		colon adenocarcinoma/melanoma	iv (1x10^7^)	(Zheng et al., 2017)
			phototherapy	colon carcinoma/breast cancer/lung carcinoma/pancreatic adenocarcinoma/cervical cancer	iv (5x10^6^)	(Yi et al., 2020)
		Flagellin B	Radiotherapy	Intracraneal sarcoma	It (1x10^6^)	(Liu et al., 2023)
SHJ2037	*ΔrelA ΔSpoT*	Cytolysin A	radiotherapy	Colon carcinoma	iv (3x10^7^)	(Liu et al., 2016)
ΔaraΔppGpp	*Δara operon ΔrelA ΔspoT*	cytolysin A		Colon carcinoma	iv (3x10^7)^	(Hong et al., 2014)
χ455O	*Δcya Δcrp Δasd*	IL-2		colon adenocarcinoma	og (4x10^7^)	(Saltzman et al., 1997)
		Immunomodulators		Breast cancer	iv (1x10^6^)	(Augustin et al., 2021)
KST0650	*Δpts Δcrr*	sATF6		colon carcinoma	ip (1x10^6^)	(Gao et al., 2020)
LPS mutants	*ΔrfaL, ΔrfaG, ΔrfaH, ΔrfaD, ΔrfaP, and ΔmsbB*			colon and renal adenocarcinoma/fibrosarcoma	iv (5x10^6^)	(Frahm et al., 2015)
YB1	*ΔaroA*. *asd* under PpepT control		5-Fluorouracilo	breast adenocarcinoma	iv (5x10^7^)	(Yu et al., 2012)
			Nanophotosensitizers	Bladder carcinoma	iv (1x10^7^)	(Chen et al., 2019a)
				hepatocellular carcinoma	iv (5x10^7^)	(Li et al., 2017, 1)
				glioblastoma	iv (8x10^7^)	(Chen et al., 2023b)
MvP728	*ΔpurD ΔhtrA*	Survivin		colon carcinoma	og (1x10^8^)	(Xu et al., 2014)
JRG4401	*ΔwaaN ΔaroA ΔpurI*	Cytolysin HlyE		mammary carcinoma	iv (1x10^6^)	(Ryan et al., 2009)
LVR01	*ΔaroC*			B-cell lymphoma	it (1x10^6^)	(Grille et al., 2014; Bascuas et al., 2018)
SL3261	*ΔaroA*	CEACAM 6 and 4-1BBL		Colorectal cancer	og (2x10^9^)	(Jin et al., 2017)
		hCD20 and HSV-TK	ganciclovir	fibrosarcoma	iv (5x10^6^)	(Massa et al., 2013)
				Melanoma	og (1x10^9^)	(Yang et al., 2008)
BRD509	*ΔaroA ΔaroD*	IFN-γ		Melanoma	sc (1x10^8^)	(Yoon et al., 2017)
		sh-INHA		colon carcinoma/melanoma	sc (1x10^8^)	(Yoon et al., 2018)
LGBM 1.1 and LGBM 1.41	*ΔihfA/ΔihfA Δasd*			Melanoma	it (1x10^7^)	(Hirsch Werle et al., 2016)
CVD 915	*ΔguaBA* mutant from S. Typhi strain Ty2.			mammary adenocarcinoma	og (1x10^9^)	(Vendrell et al., 2016)
7207	*ΔaroA*	Sh-β-catenin		colorectal adenocarcinoma	iv/og (1x10^6^)	(Guo et al., 2011)
		CtxB and PSA		mastocytoma	og (5x10^8^)	(Fensterle et al., 2008)
LH430	*ΔphoP ΔphoQ*	PD‐1-RNAi	Nifuroxazide	colon carcinoma	it (4x10^5^)	(Zhao et al., 2020)
			EZH2 inhibitors	Colorectal cancer	it (4x10^5^)	(Lv et al., 2023)
DSLpNG	*Δasd* luxCDABE operon		Doxorubicin	Breast cancer	iv (2x10^6^)	(Saltzman et al., 2018)
χ8768	*ΔphoP*	Hyaluronidases	Gemcitabine	pancreatic carcinoma	iv (2x10^6^)	(Ebelt et al., 2020)
JOL2936	*ΔtryA ΔtrypE Δasd ΔpagL ΔrfaL*	cytolysin A		Colon cancer	ip (5x10^5^)	(Aganja et al., 2023)

*S. enterica* can also be genetically modified to deliver anticancer agents (Phan et al., 2020). The release of anticancer agents into tumor cells is crucial for therapeutic efficiency. To deliver anticancer agents in the cytosol of tumor cells, anticancer agents must be transported through the bacterial envelope. Several strategies have been suggested for this purpose. For example, mutants of S*.* Typhimurium strains were designed for the *sifA* gene (Camacho et al., 2016). *sifA* gene is essential for maintaining the integrity of the vacuole that protects *S. enterica* from the action of lysosomes. *sif*A gene deletion allows *S. enterica* to escape from the endosome and locate in the cytosol (Beuzón et al., 2000); this mutation is a useful approach, even when required to deliver eukaryotic expression vectors (Beuzón et al., 2000; Camacho et al., 2016). Other strategies have been developed using a type III secretion system (T3SS). For example, the T3SS encoded by *S*. Typhimurium pathogenicity island 2 allows for efficient translocation of anticancer agents into the cytosol of tumor cells (Xiong et al., 2010; Xu et al., 2014). Both delivery strategies allow *S*. Typhimurium to function as an intracellular factory of anticancer agents that help increase the therapeutic efficacy of *S.* Typhimurium.

### 2.2 Ability to target tumor

Different investigations have shown that *S.* Typhimurium has a high affinity for tumor tissue. Although the mechanisms by which *S.* Typhimurium targets the tumor neighborhood are currently not fully elucidated, it is well established that in tumor tissue, there are chemical conditions (such as acidic pH and hypoxia) and unique or highly expressed molecules that serve as a signal for *S.* Typhimurium to detect and target the tumor microenvironment (Wojtkowiak et al., 2011; Silva-Valenzuela et al., 2016). The tumor microenvironment is typically acidic due to high glycolytic flux and substantially impairs the efficacy of chemotherapeutic drugs (Wojtkowiak et al., 2011). *S. enterica* can survive in diverse pH ranges, including the acidic pH characteristic of the tumor microenvironment (Flentie et al., 2012). The acidic pH of the tumor microenvironment inhibits immune surveillance and response, as it impairs the activity of cytotoxic cells and hinders cytokine secretion (tumor necrosis factor-alpha, interferon-gamma, IL-10, IL-12, and transforming growth factor-beta1) (Müller et al., 2000; Flentie et al., 2012). Evidence indicated that both tumor cells and acid pH induce the expression of some genes in *S.* Typhimurium. Thus, its ability to survive in acidic pH environments can help redirect immune cells to the tumor microenvironment (Broadway and Scharf, 2019).

In the tumor microenvironment, blood vessels are premature, highly irregular, and poorly organized, forming multiple regions with insufficient oxygen supply (Ryan et al., 2009). In hypoxic regions, the efficacy of conventional treatments is limited since these regions are inaccessible to chemotherapeutic agents and resistant to radiation therapy (Zhang et al., 2016). *S.* Typhimurium, as a facultative anaerobic bacterium, can overcome this problem by its ability to detect and target preferentially hypoxic regions of solid tumors, thereby inhibiting their growth rate or causing regression (Yu et al., 2012). *S*. Typhimurium YB1 can survive only hypoxic conditions and inhibits the growth of breast, liver, and metastatic tumors in nude mice, even showing more significant tumor inhibition than the VNP20009 evaluated in clinical trials (Yu et al., 2012; Li et al., 2017, 1) (**Table 2**). In addition, hypoxia-inducible promoters such as HIP-1, NirB, and FNR have been used for the targeted expression of anticancer genes at the tumor site. This way, cytotoxicity to normal tissues is reduced and increased in the tumor microenvironment (Ryan et al., 2009).

**Table 2:** Clinical trials using engineered tumor-targeting *Salmonella* Typhimurium.

Trial species	Strain	n*	Cancer type (number of patients)	Outcome or study status	Ref.
Human	VNP20009	25	Metastatic melanoma (24) metastatic renal cell carcinoma (1)	Increase of interleukin in circulation (IL-1β, TNF-α, IL-6, and IL-12); tumor colonization in three patients; No supported tumor regression	(Toso et al., 2002)
	VNP20009	4	Metastatic melanoma	No colonization or antitumor response	(Heimann and Rosenberg, 2003)
	VNP20009 expressing cytosine deaminase	3	Head and neck squamous cell carcinoma (1) Oesophageal adenocarcinoma (2)	Tumor colonization in two patients; no objective tumor regression.	(Nemunaitis et al., 2003)
	VNP20009 Expressing L-Methioninase	50	Advanced solid tumors	Not yet recruiting	(Guangzhou Sinogen Pharmaceutical Co., Ltd, 2023)
	χ4550 expressing IL-2	22	metastatic carcinoma	increased circulating NK cells. no toxicity observed	(Gniadek et al., 2020)
	χ4550 expressing IL-2	60	Metastatic pancreatic cancer	Recruiting	(Salspera, 2022)
	SS2017 expressing Tyrosine hydroxylase Phox2B, Survivin, MAGEA1, MAGEA3, and PRAME	12	Neuroblastoma	Recruiting	(Meleshko, 2022)
	CVD908ssb Expressing Survivin	18	Multiple myeloma	Recruiting	(Lulla, 2023)
					
Canine	VNP20009	41	Soft tissue sarcoma (16) Melanoma (11) Carcinoma (5) Osteosarcoma (4) Hemangiosarcoma (2) Lymphoma (2) Mast cell tumor (1)	Tumor colonization in 42% of patients; antitumor activity in 37% of patients.	(Lee et al., 2005)
	X4550 expressing IL-2	19	Appendicular osteosarcoma	No tumor colonization; no *Salmonella*-related toxicity.	(Fritz et al., 2016)

*n, number of subjects.

*S. enterica* infection induces a high infiltration of neutrophils and dendritic cells in infected tissues, limiting the *S. enterica* location to environments less accessible to immune system cells, such as hypoxic or necrotic tumor regions. Death cells in necrotic regions release molecules or nutrients such as ethanolamine, amino acids, and sugars that increase the targeting of *S. enterica* to the tumor (Zhao et al., 2006; Yu et al., 2012; Felgner et al., 2016a; Silva-Valenzuela et al., 2016; Zheng et al., 2017). Ethanolamine is a phospholipid of biological membranes used by *S. enterica* as an alternative carbon source and acts as a chemotactic agent. Amino acids required for the survival of strains with deletions in the aromatic amino acid biosynthetic pathway are also released by dying tumor cells (Zheng et al., 2017). *S*. Typhimurium strains A1-R and ΔppGpp showed a precise tumor orientation (Zhao et al., 2006; Zheng et al., 2017; Xu et al., 2022). Some *in vitro* and *in vivo* studies have shown that *S.* Typhimurium is brought into tumor cells by the serine, aspartate, and ribose/galactose receptors present on the surface of bacteria that can detect these sugars secreted by cancer cells; TAR and TRG receptors detect aspartate and ribose, respectively, promote migration to hypoxic tumor tissues (Felgner et al., 2016a). In addition, the tumor microenvironment provides conditions for *S.* Typhimurium to target the tumor and provides a suitable niche for *S*. Typhimurium to accumulate and replicate in these immune-privileged regions.

In addition to hypoxic (1% oxygen) and anoxic regions, tumors comprise regions with normal oxygen levels (8% oxygen) found near blood vessels (Casazza et al., 2014). The regions with normal oxygen levels in tumors markedly reduce the efficiency of obligate anaerobic bacteria used in cancer therapy, such as, for example, bacteria of the genus *Clostridium*, because they cannot survive or replicate in oxygen. Obligate anaerobes are not suitable for eradicating small, metastatic tumors due to their requirement for anoxic conditions, which results in bacterial colonization only in the deep area of the tumor and in the absence of bacteria at the edge of the tumor (Theys et al., 2001), leading to the recurrence of the tumor. However, facultative anaerobic bacteria, such as *Salmonella enterica*, can be distributed throughout the tissue of small (non-hypoxic), large, and metastatic (with blood vessel supply) tumors, including anoxic, hypoxic, and normoxic regions in such a way that they can be used to deliver anticancer agents throughout the tumor and not only in specific regions.

### 2.3 Preferential growth in the tumor

Systemically administered *S.* Typhimurium spreads in both tumor and healthy tissues. However, this bacterium has been shown to accumulate in tumor tissues selectively (Gao et al., 2020). Therefore, the efficacy of *S.* Typhimurium to selectively accumulate in tumors has been analyzed extensively (Clairmont et al., 2000; Gao et al., 2020). It was recently demonstrated that the highly attenuated *S.* Typhimurium strain KST0650 administered to CT26 tumor-bearing mice was eliminated from the circulation and other normal organs (spleen, lung, liver, and kidney) on days 3 and 14, respectively, while it remained in tumor tissues until day 28 after inoculation. Furthermore, this strain replicated at extraordinarily high levels in tumor tissue, reaching up to 10^9^ CFU/g of tumor tissue on the second day after inoculation, which is 1,000 to 100,000 times higher than in normal and circulating organs (Gao et al., 2020). In addition, a far-red fluorescent xenogenic pancreatic tumor model and the *S.* Typhimurium that carries luciferase cDNA made it possible to observe the distribution and accumulation of *S.* TyphimuriumVNP20009 in real-time. This dual fluorescence imaging system showed that *S.* TyphimuriumVNP20009 preferentially accumulated both in peripheral proliferative regions and in the necrotic areas of the tumor and induced apoptosis (Zhou et al., 2016).

The tumor microenvironment is an attractive target for *S. enterica*, probably because it provides privileged conditions that favor bacterial colonization, and this preferential accumulation elicits anticancer effects (Zhou et al., 2016; Mi et al., 2019). *S.* Typhimurium A1 selectively accumulates in tumor tissue (Zhao et al., 2005). This auxotrophic bacterium takes advantage of nutrients in the tumor tissue for survival, especially the essential amino acids leucine and arginine. Selective accumulation of strain A1 resulted in complete regression of subcutaneous tumor within 15-26 days after the initiation of treatment (Zhao et al., 2005). This strain was potentiated to increase tumor colonization by passages in colon tumor-bearing mice, resulting in the isolation of A1-R. In addition, A1-R showed an enhanced ability to grow preferentially in tumor tissue, as well as potent anticancer activity in various primary and metastatic murine tumor models (Hayashi et al., 2009; Liu et al., 2010; Yam et al., 2010). These findings have evidenced the potential use of *S. enterica* alone or as a delivery vector for treating cancer.

Conventional treatments often damage normal healthy cells and tissues; however, using *S. enterica* can overcome this limitation. The specificity accumulation of *S. enterica* in the tumor microenvironment can allow the administration of more toxic molecules directed to the tumor without side effects in healthy adjoining tissues (Zheng et al., 2017; Bascuas et al., 2018). To enhance the therapeutic effects of *S.* Typhimurium and overcome the limitations associated with the administration of interferon γ (IFN-γ) for melanoma treatment, a strain capable of secreting IFN-γ into the cytosol of host cells was constructed using the *S*. Typhimurium BRD509 T3SS. IFN-γ is a promising anticancer agent; however, its use in cancer therapy has certain limitations, such as the cytotoxicity associated with its direct administration and short half-life. *S*. Typhimurium BRD509 secreting IFN-γ showed selective growth in B16F10 mouse melanoma when administered by three routes of administration (intravenous, oral, and subcutaneous), and subcutaneous treatment significantly inhibited tumor growth. Furthermore, no histopathological signs of damage or adverse effects were found in normal tissues; these results show the ability of *S.* Typhimurium to preferentially attack cancer cells without generating significant toxicity to normal tissues (Yoon et al., 2017). Similar results were obtained recently (Xu et al., 2022).

### 2.4 Intratumoral penetration

Systemic administration of chemotherapeutic drugs is often ineffective due to the inability to penetrate poor vasculature regions and increasing amounts of the extracellular matrix characteristic of the tumor microenvironment (Agarwal et al., 2013; Zhang and Forbes, 2015). Chemotherapeutic agents enter the tumor tissue through the vasculature. Therefore, in regions with large intercapillary distances or slow blood flow, penetration tends to be low or null, which prevents chemotherapeutics from diffusing deep into tumors and reaching efficient concentrations (Zhang and Forbes, 2015). The extracellular matrix acts as a biophysical barrier against cytotoxic therapies, and in solid tumors, it is mainly composed of high levels of elastin, hyaluronan, fibrillar collagens, fibronectin, and laminins (Ebelt et al., 2020). It has been shown that in tumors, the deposition of extracellular matrix by tumor cells and microblasts associated with cancer is excessive, resulting in the ineffectiveness of systemic therapy in reduced penetration of chemotherapeutics (Henke et al., 2019; Zargar et al., 2019). Some drugs capable of degrading components of the extracellular matrix have been proposed to improve the administration of chemotherapeutics. However, their use has been limited due to the systemic degradation of the extracellular matrix of healthy tissues (Ebelt et al., 2020). For instance, the administration of hyaluronidase has shown clinical benefits in patients with tumors that express high levels of hyaluronic acid, such as pancreatic ductal adenocarcinoma. However, there is some concern about the increased risk of adverse effects due to the degradation of the extracellular matrix in healthy tissues (Doherty et al., 2018).

Metabolically active *S.* Typhimurium can overcome this limitation due to its ability to penetrate through poor vasculature regions and reach regions not easily accessible to the drugs administered systemically (Zhang and Forbes, 2015; Yoon et al., 2018). Previous work used attenuated *S.* Typhimurium for strictly regulated inducible expression of the bacterial hyaluronidase in orthotopic tumors that express high levels of hyaluronic acid. *S.* Typhimurium χ8768 was administered systemically to mice with pancreatic tumors penetrated and reached tumor-specific colonization two days after inoculation. Once there, hyaluronidase expression was activated by a single injection of arabinose to degrade tumor-specific hyaluronic acid and reduce the risk of degrading the extracellular matrix of healthy tissues. Hyaluronidase expressed on the surface of *S.* Typhimurium degraded tumor-specific hyaluronic acid and significantly improved the efficacy of the chemotherapeutic gemcitabine; Additionally, the degradation of hyaluronic acid resulted in a greater penetration of *S.* Typhimurium throughout the tumor (Ebelt et al., 2020).

Inherently mobile attenuated strains of *S. enterica* penetrate away from the tumor vasculature, accumulate, colonize, and inhibit tumor growth (Ganai et al., 2011; Raman et al., 2019). *S. enterica* motility is crucial for intratumoral penetration (Yu et al., 2012; Zhang and Forbes, 2015). *S.* Typhimurium can penetrate deeper into the tumor microenvironment and accumulate to considerably higher concentrations because of its self-propelling properties (Toley and Forbes, 2012; Thornlow et al., 2015; Li et al., 2017). The analysis of the swimming speed in a microfluidic device showed that *S.* Typhimurium (SL1344 and VNP20009) was more mobile than *E. coli* (DH5α and K12); the faster swimming strains showed more significant accumulation and deep intratumoral penetration (Toley and Forbes, 2012). Mobility within a bacterial strain is heterogenic; fractions of non-mobile, minimally mobile, and highly mobile individuals exist. These fractions were quantified in *S.* Typhimurium, and it was found that less than 15% of the total population constituted the fraction of highly mobile individuals actively penetrating the tumor tissue (Zhang and Forbes, 2015). The highly mobile fraction of *S.* Typhimurium was isolated; this fraction showed improved penetration and accumulation compared to unselected controls (Thornlow et al., 2015). These and other results strongly suggest that tumor penetration and intratumoral accumulation are mechanisms influenced by motility (Thornlow et al., 2015; Raman et al., 2019).

Previous studies have demonstrated a regulatory link between flagella-dependent motility and intracellular invasion through the master regulator *flhDC* (Elhadad et al., 2015; Raman et al., 2019). Deletions of flagellar genes decrease intracellular invasion of *S.* Typhimurium, and strains with greater mobility showed increased intracellular invasion (Elhadad et al., 2015). The *flhDC* complex controls the *fliZ* motility regulator, and the latter positively regulates cellular levels of the *hilD* transcription factor, which controls the transcription of the genes that make up T3SS and intracellular invasion (Raman et al., 2019). A recent study demonstrated the relationship between motility, intracellular bacterial density, and tumor colonization; *S.* Typhimurium actively penetrated the tumor masses after overexpression of the master regulator *flhDC, S.* Typhimurium swam much faster than the uninduced control (Raman et al., 2019). The overexpression of *flhDC* promoted intracellular accumulation of *S.* Typhimurium and tumor colonization *in vitro*. Induction of *flhDC* after initial penetration increased invasion-dependent T3SS and subsequently increased intracellular bacterial density; colonization was also enhanced by *flhD* overexpression due to increased retention of bacteria in tumors (Raman et al., 2019). The efficacy of bacterial immunotherapy could be improved with this inducer to drive more significant bacterial colonization and administration of payloads.

## 3 *Salmonella enterica* tumor suppression mechanisms

**Figure 2:**
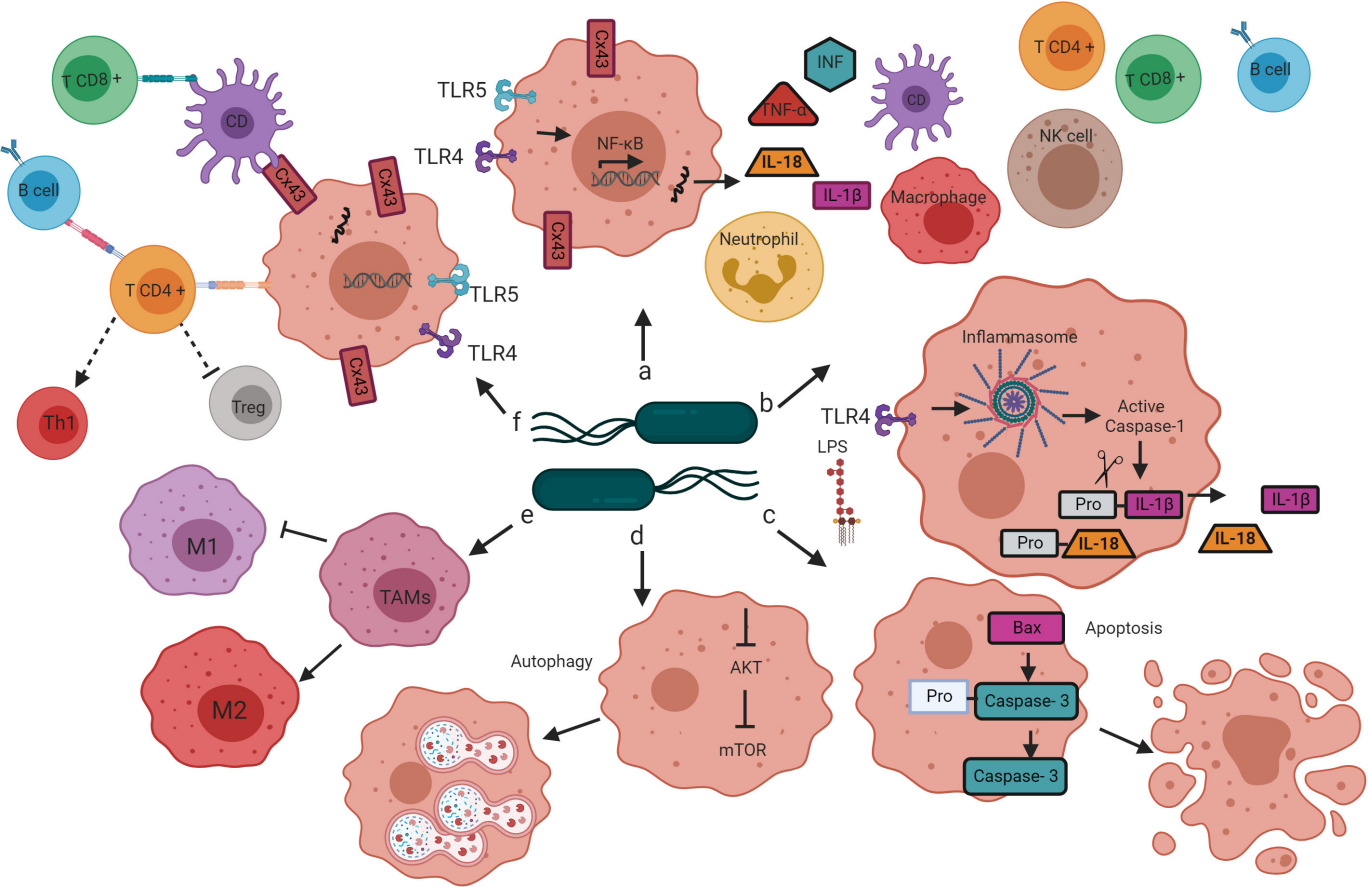
Mechanisms by which bacteria target tumors. *Salmonella*’s colonization of the tumor microenvironment induces the activation of the innate and adaptive immune response through various mechanisms that lead to tumor regression. **(A)** The interaction of *Salmonella* LPS and flagellin with the TLR4 and TLR5 receptors, respectively, leads to the activation of the transcription factor NF-κB, which induces the transcription of proinflammatory cytokines (TNF-alpha, INF, IL-1b, and IL-18) that promote infiltration of dendritic cells, neutrophils, macrophages, NK cells, T cells and B cells in the tumor microenvironment. **(B)** *Salmonella* LPS activates the inflammasome pathway, resulting in caspase-1-dependent secretion of IL-1B and IL-18 in the tumor microenvironment. **(C)** *Salmonella* increases the expression of the Bax protein and caspase-3 in tumor cells, directing cellular changes characteristic of apoptosis. **(D)** *Salmonella* activates autophagy in tumor cells by down-regulation of the AKT/mTOR signaling pathway. **(E)** *Salmonella* reprograms the polarization of TAMs from a phenotype that contributes to tumor development (M2) to a phenotype that directs the elimination of cancer cells (M1). **(F)** *Salmonella* increases the expression of Cx43, a protein that forms the jap junctions, which are essential for cross-presenting tumor antigenic peptides by DC to CD8 + T cells. *Salmonella* also activates CD4 + T cells, once active, these cells direct the activation and differentiation of B cells, the B cells, in turn, contribute to the polarization of CD4 + T cells to the Th1 phenotype and the decrease in the population of Tregs.

Infection with attenuated *S. enterica* can cause tumor regression or inhibition through several mechanisms (Avogadri et al., 2005; Kaimala et al., 2014). Some studies have reported that *S.* Typhimurium has direct tumoricidal activity (Avogadri et al., 2005; Li et al., 2012; Hirsch Werle et al., 2016); however, most studies point toward activating the innate and adaptive immune response as the primary mechanism for *S.* Typhimurium to destroy solid tumors and metastases (Chang and Lee, 2014) (**Figure 2**). Next, we review the alterations that the treatment of tumors with *Salmonella enterica* induces in the immune system, leading to tumor regression in animal models. Understanding these mechanisms could help explain the limited efficacy of previous clinical trials and design improved strategies for translational application of *Salmonella enterica-*based cancer immunotherapy.

### 3.1 *Salmonella enterica* induces the activation of the immune system

The tumor microenvironment is immunologically privileged because it escapes from immune surveillance and prevents infiltration of immune cells (Zheng and Min, 2016). The administration of *S. enterica* increases the antigenicity of low antigenic tumors by activating Toll-like receptors (TLRs) and triggering the infiltration of antigen-presenting cells (macrophages and dendritic cells) and T cells (Kaimala et al., 2014). The pathogen-associated molecular patterns (PAMPs) present on the surface of *S. enterica* activate TLRs expressed by innate immune cells (Phan et al., 2020). Some studies have described the therapeutic effect of the activation of TLR5 by flagellin (de Melo et al., 2015); TLR5 recognizes the flagellum on the surface of macrophages, monocytes, CD4 T cells, and dendritic cells. A recent study showed that the *Salmonella* Typhimurium VNP20009 flagellum is required to induce a significant antitumor response; Flagellum lacking strains (Δ*flhD* or Δ*fliE*) were unable to reduce the size of melanoma tumors, which suggests that the flagellum plays a crucial role in the antitumor response mediated by *Salmonella* Typhimurium (Chen et al., 2021).

Similarly, it was shown that *S. enterica* serotype Choleraesuis inhibits tumor growth in the murine model and induces the recruitment of innate immune response cells through TLR4 signaling (Lee et al., 2008). Activation of TLR4 by lipopolysaccharide results in translocation of nuclear factor κB (NF-κB) into the nucleus, where molecular signals that lead to the expression of genes that encode proinflammatory cytokines such as interleukin-1β (IL-1β), interleukin-18 (IL-18), interferon-γ (IFN-γ), and necrosis factor-α(TNF-α). Secreted proinflammatory cytokines promote infiltration of dendritic cells, natural killer (NK) cells, macrophages, neutrophils, T and B cells, transforming the immunosuppressed tumor microenvironment into immune-activated (Kaimala et al., 2014; Kim et al., 2015; Li et al., 2020b).

The inflammasome regulates IL-1β and IL-18 production (Phan et al., 2015). The macrophage inflammasome is a cytosolic macromolecular complex that assembles to recruit and activate the cysteine protease caspase-1, which cleaves the pro-IL-1β and pro-IL-18 proinflammatory cytosines to their active form (Rathinam and Fitzgerald, 2016). Previous studies demonstrated that the antitumor activity of attenuated *S*. Typhimurium ΔppGpp involves the activation of the inflammasome (Phan et al., 2015). The analysis of the mRNA expression of key molecules for the activation of the inflammasome (IPAF and NLRP3) in mice treated with *S.* Typhimurium ΔppGpp and with *E.* coli MG1655 showed a significant elevation of these molecules when treated with *S.* Typhimurium ΔppGpp. The inflammasome is activated by the interaction of *S.* Typhimurium LPS with macrophages and ATP released by damaged cancer cells. Activating the inflammasome by *S.* Typhimurium significantly increased IL-1β release in tumors, inducing cell death (Phan et al., 2015).

Cell death pathways are inactive or altered in high-grade malignancy tumors (Hanahan and Weinberg, 2011). However, substantial evidence indicates that *S.* Typhimurium can induce apoptosis of tumor cells by multiple mechanisms, including accumulation in the tumor microenvironment, competition for food, toxin secretion, and recruitment of cells from the immune system (Wang et al., 2018; Li et al., 2020b). Treatment with *S.* Typhimurium VNP20009 caused apoptosis by increasing the expression of caspase-3 and Bax protein in two models of murine cancer (acute leukemia and pancreatic ductal adenocarcinoma), leading to tumor regression (Zhou et al., 2016; Li et al., 2020b). In addition to apoptosis, *S.* Typhimurium can induce cell death of cancer cells by autophagy (Lee et al., 2014; Wu et al., 2022). Under normal conditions, autophagy occurs in cells at low basal levels and only increases in response to stress conditions such as nutrient deficiency (Tu et al., 2016). However, in tumor cells, the activation of autophagy is altered (Hanahan and Weinberg, 2011). In the murine model, attenuated *S.* Choleraesuis controlled tumor growth by inducing autophagy by down-regulation of the AKT/mTOR signaling pathway (Lee et al., 2014; Wu et al., 2022). The activation of cell death pathways, such as apoptosis and autophagy, is involved in the antitumor activity of *S. enterica.*

*S.* Typhimurium also induces tumor growth suppression from macrophages (Kaimala et al., 2014); the macrophages are the main niche of this bacterium (Yang et al., 2018). Macrophages are heterogeneous cells that play a central role in the antitumor efficacy of *S. enterica*. Depending on the polarization signals received, these cells can express different and complex phenotypes (Perrotta et al., 2018). Macrophages M1 and M2 are the major phenotypes of macrophages and constitute the two extremes of the wide variety of macrophage phenotypes (Yang et al., 2018); these phenotypes have different effect on tumors (Yang et al., 2018). For example, M1 or classically activated macrophages respond to PAMPs and orchestrate pro-inflammatory and antitumor immune responses that eliminate cancer cells (Kaimala et al., 2014). In contrast, M2 macrophages, also known as alternatively activated, are polarized by anti-inflammatory molecules Interleukin-4 (IL-4) and Interleukin-13 (IL-13) and are characterized by supporting tumor growth angiogenesis, metastasis, and resistance to chemotherapeutics (Perrotta et al., 2018; Di Mitri et al., 2019). Macrophages found in the stroma of solid tumors are called tumor-associated macrophages (TAMs). TAMs are the most abundant population in the tumor microenvironment and are principally polarized to the M2 phenotype (Nava Rodrigues et al., 2018).

The phenotype of differentiated TAMs can change through exposure to different stimuli (Flentie et al., 2012; Silva-Valenzuela et al., 2016). For example, it was recently reported that *S.* Typhimurium could reprogram polarized TAMs from phenotype M2 to M1 (Yang et al., 2018; Zhang et al., 2023). When the M2 macrophages were co-cultured with *S.* Typhimurium YB1 under hypoxic conditions for two hours, more than 50% of the M2 macrophages phagocytosed the bacteria. Furthermore, after co-culturing with *S.* Typhimurium, M2 macrophages activated by IL-4 treatment showed increased human leukocyte antigen-D antigen (M1 macrophage phenotype marker) and reduced expression of CD206 (phenotype marker of macrophages M2) (Yang et al., 2018). These findings suggest the efficacy of *S.* Typhimurium YB1 in reprogramming the M2 to M1 phenotype and probably show one of the strategies by which *S.* Typhimurium YB1 inhibits tumor growth. Reprogramming of TAM M2 to M1 using *S.* Typhimurium suggests the potential use of this bacterium to reduce a cell population that contributes to tumor progression by increasing the cell population that kills tumor cells.

The recruitment of the innate immune system cells is the primary mechanism by which *S.* Typhimurium causes the regression of tumors (Bascuas et al., 2018). Proinflammatory cytokines released in response to *S.* Typhimurium accumulation in the tumor microenvironment recruit and promote dendritic cell (DC) maturation (Kim et al., 2015; Li et al., 2020b). DC maturation also requires gap junctions that allow intracellular communication and transfer of pre-processed antigenic peptides (Kiszner et al., 2019). The gap junctions comprise six connexin proteins (Cx), including Cx43(Kazan et al., 2019; Kiszner et al., 2019). It has been documented that several tumors have a low expression of Cx43, and even this protein is lost during tumor progression. *S.* Typhimurium can enhance the expression of Cx43 in melanoma cells (human and murine) (Saccheri et al., 2010) and promote the presentation of antigenic peptides of tumor cells by DC (Mehner et al., 2014). DCs target lymph nodes to present antigenic peptides by class I major histocompatibility complex to cytotoxic T lymphocytes (CTL) and NK cells (Saccheri et al., 2010). Once activated, these cells can trigger the destruction of tumor cells (Kim et al., 2015; Chen et al., 2019b).

The *S.* Typhimurium-mediated antitumor immune response is limited to myeloid and includes lymphoid cells (Zhao et al., 2005). Therefore, it has been proposed that T and B cells are necessary for the antitumor activity of *S.* Typhimurium (Lee et al., 2011a; Lee et al., 2011b). Previous studies in T-cell deficient mice indicate that these cells play a crucial role in the antitumor effect of *S.* Choleraesuis. Indeed, the systemic administration of *S.* Choleraesuis in tumor-bearing mice showed that in the absence of T cells, the antitumor effect of *S.* Choleraesuis is less efficient (Lee et al., 2011b). Several studies using *S.* Typhimurium to treat murine tumors have also supported the crucial role of T cells for antitumor efficacy. For example, treatment with *S*. TyphimuriumVNP20009 in mice with acute myeloid leukemia increased NK, Th1, and CD8+ cells and significantly prolonged survival (Li et al., 2020b).

Similarly, the antitumor immune response activation, achieved through *S*. Typhimurium LVR01, prolonged survival and reduced tumor growth in a murine lymphoma model through intratumoral recruitment of CD8^+^, NK, helper T cells 1 (Th1), and neutrophils (Grille et al., 2014). In addition, an enhanced effect was observed when mice with lymphoma were treated with *S*. Typhimurium LVR01 and interleukin-2 (IL-2) (Grille et al., 2013). The combination treatment increased NK and T cell infiltration in lymphoma tumors, probably due to the IL-2 activating these cells (Grille et al., 2013). These results strongly suggest that *S.* Typhimurium alone or IL-2 modulates the T cell-mediated antitumor response activation.

The induction of tumor regression by *S.* Typhimurium has been associated with differentiating CD4^+^ T cells into Th1 and decreasing the population of regulatory T cells (Tregs) (Jin et al., 2017). Indeed, previous work has demonstrated that Treg cells contribute to maintaining an immunosuppressed tumor microenvironment (Kim et al., 2015). *S.* Typhimurium can trigger tumor regression by decreasing the population of Tregs cells through LPS and Braun’s lipoprotein, as demonstrated in a previous study (Liu and Chopra, 2010). The *msbB* mutation of *S.* TyphimuriumVNP20009 probably significantly influenced the low tumor regression observed in clinical trials with this strain (Toso et al., 2002). A recent study showed that *S.* Typhimurium inhibited the development of colorectal tumors in a rat model through polarization to the Th1 phenotype and inhibition of polarization to the Th2 and Th17 phenotypes (Jin et al., 2017). The polarization of the Th1 phenotype is mediated by TLR4 MyD88 signaling activated by *S.* Typhimurium LPS and requires the activity of B cells (Lee et al., 2011a). Finally, *S.* Typhimurium induced T cell differentiation into Th1 and IFN-γ production by Th1 cells in wild-type mice but not in mice incapable of producing B cells (Lee et al., 2011a). Th1 cells produce high levels of IFN-γ, a cytokine that acts as an effector in CTL activity and is necessary to direct an effective immune response that promotes tumor suppression (Lee et al., 2008; Jin et al., 2017).

Although the role of T cells in *S.* Typhimurium-mediated tumor immunotherapy has been extensively studied and used to enhance the efficacy of *S.* Typhimurium in suppressing tumors (Lee et al., 2008; Lee et al., 2011b; Jin et al., 2017), the contribution of B cells in this process is poorly understood. However, the available evidence suggests that B cells are essential for *S.* Typhimurium-mediated antitumor activity. B cells can act as antigen-presenting cells and contribute to T-cell activation by cross-presentation (de Wit et al., 2010; Kim et al., 2015; Vendrell et al., 2016). For example, treating murine tumors with *S*. Typhimurium ΔppGpp induced tumor suppression from infiltrating leukocytes, T cells, and B cells (Kim et al., 2015; Xu et al., 2022). Another study in a murine model showed that attenuated *S.* Choleraesuis was more efficient in inhibiting tumor growth in B-cell deficient mice than in wild-type mice; however, it significantly decreased the survival of B-cell deficient mice. Moreover, a higher bacterial load and expression of inflammatory cytokines was found in the blood, liver, and spleen of mice deficient in B cells than in wild-type mice (Lee et al., 2011a); These data indicate that B cells are essential for the antitumor effect of *S. enterica*, but more studies are needed to elucidate its role in *S. enterica*-mediated tumor immunotherapy.

The mechanisms that explain the efficacy of *S. enterica*-mediated immunotherapy are complex and are not yet fully understood. However, accumulated evidence attributes its effectiveness to inducing proinflammatory cytokine production and activating innate and adaptive immune system cells. In addition, activated immune cells lead to the death of tumor cells and induce changes in the tumor microenvironment, which changes from immunosuppressed to immunocompetent to exert its anticancer effects. *S.* Typhimurium can also cause tumor progression inhibition by suppressing angiogenesis and metastasis (Miwa et al., 2014; Tu et al., 2016; Vendrell et al., 2016; Yi et al., 2020).

### 3.2 *S.* Typhimurium inhibits angiogenesis and delays metastasis

The progression of some tumor types is well marked by the migration of cancer cells from the primary site of the tumor to different sites in the body through the blood vessels. Blood vessels supply nutrients and oxygen necessary for the uncontrolled growth and survival of cancer cells (Saman et al., 2020); therefore, forming blood vessels from existing endothelial-lined vessels is essential for tumor growth (Quintero-Fabián et al., 2019; Saman et al., 2020). The inhibition of angiogenesis is one of the promising therapies, and the United States Food and Drug Administration (FDA) has approved therapeutic strategies for treating cancer, including drugs that inhibit angiogenesis (Cook and Figg, 2010). However, antiangiogenic therapy has many side effects and has not shown the expected effectiveness (Carmeliet and Jain, 2011). Numerous studies have demonstrated that *S.* Typhimurium can inhibit angiogenesis and delay metastasis (Miwa et al., 2014; Tu et al., 2016; Vendrell et al., 2016; Yi et al., 2020).

*S. enterica* can inhibit the expression of angiogenic activators such as vascular endothelial growth factor (VEGF). VEGF has been described as a potent angiogenic agent during tumor growth because it can induce endothelial cell growth (Saman et al., 2020). Studies demonstrated that *S.* Choleraesuis suppressed tumor angiogenesis by downregulating VEGF expression. B16F10 and 4T1 tumor cells treated with *S.* Typhimurium drastically decreased VEGF expression by inhibiting hypoxia-induced factor 1α, thus suppressing the proliferation of cancer cells (Tu et al., 2016). These results were confirmed *in vivo* when systemically administered *S.* Choleraesuis decreased VEGF levels in mice bearing B16F10 and 4T1 tumors; in addition, the *Salmonella*-treated mice tumors were smaller and less vascularized than the untreated mice (Tu et al., 2016). Angiogenesis is essential for tumor growth and for cancer cells to migrate and metastasize to distant sites. In addition, the blood vessels provide the primary route for cancer cells to escape from the primary site of the tumor (Wang et al., 2014).

In addition to angiogenesis, matrix metalloproteinases (MMP) can contribute to tumor cell migration. Tumor cells require the proteolytic action of MMP to degrade the extracellular matrix (Quintero-Fabián et al., 2019). Angiogenic agents, including VEGF, can activate MMPs, promoting angiogenesis and metastasis (Gupta et al., 2013; Quintero-Fabián et al., 2019). MMPs are endopeptidases that bind to zinc and participate in remodeling the extracellular matrix (ECM) by degrading its components. Excessive degradation of ECM components, including the tumor surface, promotes tumor cell migration (Quintero-Fabián et al., 2019). Studies have revealed that in various types of cancer, the expression of MMP-9 is elevated and contributes to metastasis. MMP exerts its proteolytic activity on the components of the ECM, thus promoting the migration of cancer cells (Gupta et al., 2013; Mehner et al., 2014). *S.* Choleraesuis inhibits the migration of tumor cells through decreased MMP-9 expression in melanoma and lung carcinoma murine cells (Tsao et al., 2018). The AKT/mTOR signaling pathway was found to control the decrease in MMP-9 expression induced through *S.* Typhimurium. *In vivo*, it was also possible to observe the decrease in MMP-9 activity, and it was related to the decrease in metastatic nodules in the groups administered with *S.* Choleraesuis (Tsao et al., 2018). Similarly, *S.* Choleraesuis reduces metastasis by downregulating epithelial cell adhesion molecules *via* the AKT/mTOR signal pathway (Yen et al., 2023). All these results suggest that treating tumors with *S. enterica* can decrease metastasis.

### 3.3 *S.* Typhimurium induces ferroptosis

**Figure 3:**
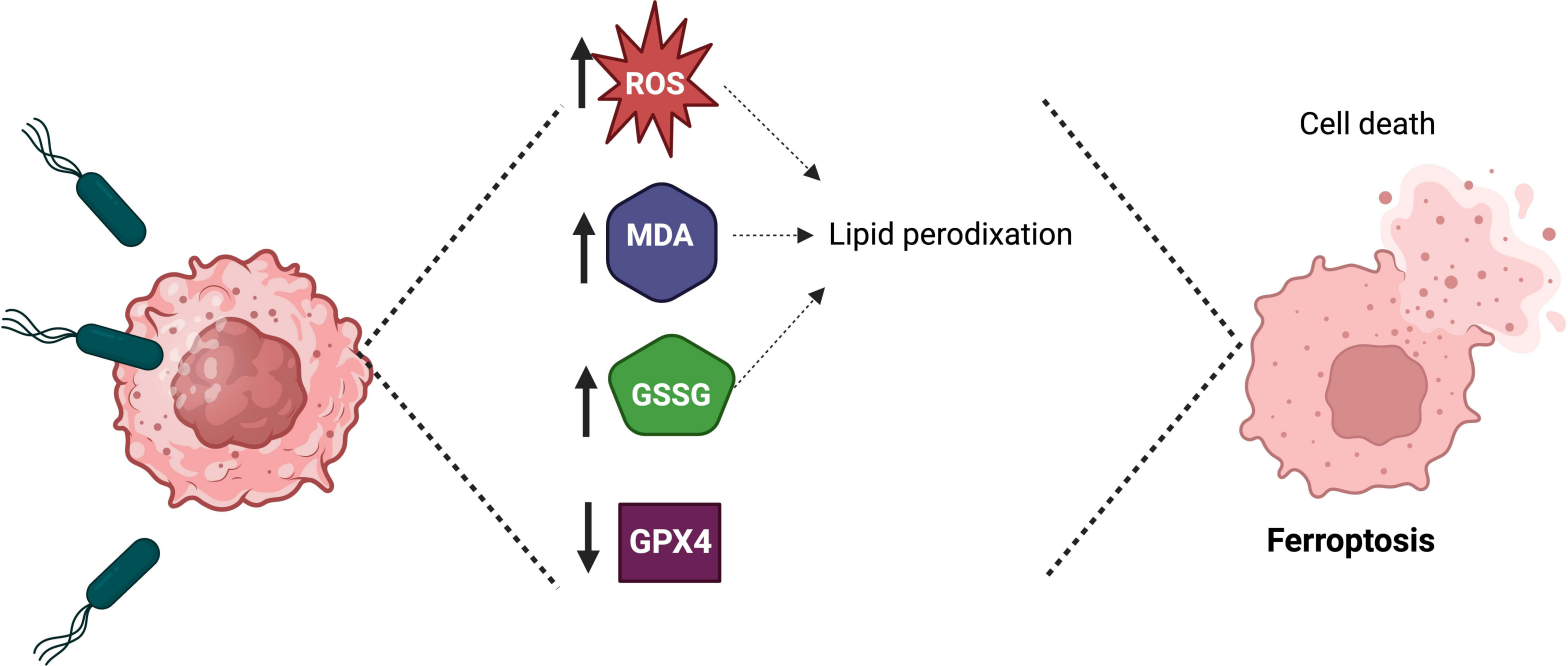
*Salmonella* Typhimurium induces ferroptosis to suppress tumor growth. The treatment of tumor cells with *Salmonella* Typhimurium increases the production of lipid reactive oxygen species (ROS), malondialdehyde (MDA), and oxidized glutathione (GSSG), as well as *in vivo* the treatment of tumor-bearing mice with *Salmonella* Typhimurium decreases the expression of glutathione peroxidase-4 (GPX4), which leads to ferroptosis.

Recently, it was demonstrated *in vitro* and *in vivo* that *S.* Typhimurium could induce ferroptosis to inhibit tumor growth (**Figure 3**) (Chen et al., 2023b). Ferroptosis is a form of cell death characterized by iron accumulation in the cell and lipid peroxidation (Li et al., 2020a). Ferroptosis can be activated through the blockade of glutathione peroxidase GPX4 (the intracellular enzyme that counteracts the oxidation of lipids in the membrane). The infection of glioma cells (U87 and U251) with *S.* Typhimurium increased three critical hallmarks of ferroptosis: lipid reactive oxygen species (ROS), malondialdehyde (MDA) and oxidized glutathione (GSSG), compared with that in the control group. A murine glioma model demonstrated that *S.* Typhimurium could induce the tumor death cell by activating ferroptosis and reducing glutathione peroxidase-4 expression (Chen et al., 2023b). These results suggested that *S.* Typhimurium suppresses murine glioma growth through ferroptosis induction. Future research could elucidate whether *S.* Typhimurium activates ferroptosis in tumors other than glioma.

## 4 *S.* Typhimurium as a vector to deliver anticancer agents

Despite numerous studies on the efficiency of *S. enterica* in inhibiting tumor growth in tumor-bearing mouse models, *S. enterica* alone is often insufficient to eliminate tumors. To enhance its therapeutic effects, *S. enterica* has been used as a vector for delivering anticancer agents (Yoon et al., 2017). The delivery of anticancer agents must be strictly controlled to ensure the safety and efficacy of the treatment (Camacho et al., 2016). The ability of *S.* Typhimurium to specifically target the tumor contributes to the targeted delivery of anticancer agents. However, inducible promoters have been proposed to ensure tight control of anticancer agent delivery. For example, hypoxia-inducible promoters (HIP-1, NirB, and FNR), L-arabinose promoter (pBAD), and tetracycline promoter (pTet) have been used (Ryan et al., 2009). *S.* Typhimurium secretion systems have also been used to efficiently deliver therapeutic agents into the cytosol of cells (Xu et al., 2014; Camacho et al., 2016; Clark-Curtiss and Curtiss, 2018). Currently, the most widely used anticancer agent delivery strategies are: (I) Delivery of genetic material for the eukaryotic expression of antitumor agents or the expression of RNA interference (RNAi) (Lee et al., 2005; Yang et al., 2008; Guo et al., 2011; Yoon et al., 2017; Yoon et al., 2018); (II) Delivery of cytotoxic agents to induce apoptosis of tumor cells (Camacho et al., 2016; Yang et al., 2016); (III) Delivery of immunomodulators to enhance the antitumor immune response (Saltzman et al., 1996; Nishikawa et al., 2006; Bereta et al., 2007; Fensterle et al., 2008; Loeffler et al., 2008; Massa et al., 2013; Schmitz-Winnenthal et al., 2015; Park et al., 2016; Augustin et al., 2021); and (IV) Delivery of Prodrug converting enzymes (King et al., 2002; Nemunaitis et al., 2003; Friedlos et al., 2008).

### 4.1 Delivery of genetic material

Most studies using *S. enterica* to deliver anticancer agents use the bacteria’s machinery to express the molecules of interest. However, this approach, in some cases, has limitations. For example, when the molecule of interest is a mammalian protein with anticancer activity. Mammalian proteins expressed by bacteria often lack biological activity due to the absence of post-translational modifications and correct folding (Tokmakov et al., 2012). Therefore, the administration of eukaryotic expression vectors by bacteria, a process known as bactofection, eliminates this problem because it allows the obtaining of biologically active proteins (Yoon et al., 2018; Johnson et al., 2019). In bactofection, intracellular bacteria deliver genes to a target cell for protein expression (Johnson et al., 2019). *S.* Typhimurium SL3261 was recently used to deliver genes encoding for carcinoembryonic antigen-related cell adhesion molecule 6 (CEACAM6) and 4–1BB ligand in (4–1BBL) in colon tumor-bearing mice. The bactofection of these genes with *S.* Typhimurium SL3261 inhibits the growth of colorectal tumors (Jin et al., 2017).

Recently, an *S.* Typhimurium high attenuated strain has been used to deliver the p53 gene to human bladder adenocarcinoma *in vitro* using the cell lines for human bladder carcinoma 5637 and transitional cell papilloma RT4 (Pérez Jorge et al., 2022). Both cells express loss of function in the tumor suppressor p53, one of the most frequent mutant genes in bladder cancer (Liao et al., 2021) and other cancers (Duffy et al., 2022). Pérez Jorge et al. (2022) observed that transfected cells express the p53 transgene and that such expression drives cancer cell loss of viability. These results corroborate that observed by a previous study using a *S.* Typhimurium strain with a different genetic background (Jiang et al., 2013).

Furthermore, *S.* Typhimurium has been reported to deliver RNAi of genes that drive tumorigenesis, known as oncogenes (Yoon et al., 2018). Oncogenes encode transcription factors that promote the proliferation and metastasis of tumor cells. In multiple types of cancer, some oncogenes are highly expressed. Decreased expression of oncogenes in tumor cells mediated by small strands of RNA complementary to the mRNA of oncogenes can restrict tumor growth (Chen et al., 2018b). Several drugs based on RNAi targeting oncogenes mRNA have been proposed. However, efficient administration has been a critical limitation (Chen et al., 2018b). *S.* Typhimurium has shown efficient delivery of RNAi from different oncogenes (Yang et al., 2008; Guo et al., 2011; Yoon et al., 2018). *S.* Typhimurium was modified to deliver short hairpin RNA specifically targeted to the alpha subunit of inhibin (sh-INHA) from a eukaryotic expression plasmid. The results showed that sh-INHA delivery by the bacterium significantly decreased INHA expression in tumor-bearing mice (colon CT26 and melanoma B16F10) and inhibited tumor growth (Yoon et al., 2018).

### 4.2 Delivery of cytotoxic agent

The delivery of cytotoxic agents by *S.* Typhimurium is the most used strategy to improve their antitumor activity. Inducible promoters to avoid or reduce toxic effects in healthy tissue are often used to control the expression of cytotoxic agents. For example, *S.* Typhimurium was designed to express a mutant of the Fas-associated protein with a C-terminus truncated death domain (N-FADD) from the hypoxia-induced NirB promoter in tumor tissues. *S.* Typhimurium expressing N-FADD showed remarkable tumor regression. The primary mechanism involved in the regression observed in this study was the activation of the caspase-3-dependent apoptotic pathway (Yang et al., 2016). Similar results were obtained when *S.* Typhimurium was used to deliver ClyA under a promoter that responds to low oxygen concentrations and HlyE under the control of an arabinose-inducible promoter (Ryan et al., 2009; Bhattacharya et al., 2020).

Other studies have designed tightly regulated systems to deliver cytotoxic agents to tumor tissues (Camacho et al., 2016). For example, the cytotoxic peptide Cp53 was released into the cytosol of tumor cells by a strain of *S.* Typhimurium designed by genetic engineering from the combination of three regulatory elements. The first element is an inducible autolysis system, which, in response to anhydrotetracycline, liberates the content of *S.* Typhimurium by lysis induction; the second element is an aspirin-induced salicylate cascade system. This system will only allow the production of the therapeutic agent in the presence of aspirin. Finally, the third element is deleting the *sifA* gene from the bacterial chromosome; this mutation allows *S.* Typhimurium to escape from the vacuolar environment to be located in the cytosol. The designed strain was initially induced to produce Cp53 within tumor cells; after bacterial lysis, it was induced to release the Cp53 peptide. This strain was shown to be efficient in inducing apoptosis of tumor cells (Camacho et al., 2016).

### 4.3 Delivery of immunomodulators

*S.* Typhimurium has been engineered as a delivery vehicle to immunomodulators such as cytokines, ligands, and antigens (Lee et al., 2005; Zhao et al., 2020). Cytokines can induce apoptosis of tumor cells. However, the short half-life of these molecules may limit this approach to cancer therapy (Donohue and Rosenberg, 1983). The intrinsic ability of *S.* Typhimurium to colonize and replicate in tumors can address this limitation. IL-18 and IL-2 delivery by *S.* Typhimurium resulted in the regression of primary subcutaneous and metastatic tumors associated with increased intratumoral cytokines accumulation of CTL, NK cells, and granulocytes (Saltzman et al., 1996; Loeffler et al., 2008). Similarly, other authors demonstrated the expression of the antigens Survivin and NT-ESO-1 by *S.* Typhimurium. The expression of these antigens results in T cell-dependent tumor regression (Nishikawa et al., 2006; Xu et al., 2014). Therefore, immunomodulators administered by *S.* Typhimurium can enhance the antitumor immune response and facilitate the death of tumor cells.

Tumor regression and improved tropism were observed when arginine-glycine-aspartate (RGD) peptide was expressed on the surface of *S.* Typhimurium ΔppGpp (Park et al., 2016; Liang et al., 2022). The RGD peptide is a ligand that binds to integrin alpha v beta 3 (αvβ3). In cancer cells, the αvβ3 integrin is overexpressed (Wang et al., 2019). *S.* Typhimurium expressing RGD established stable contact with tumor cells by the union between the RGD peptide and the αvβ3. Furthermore, *S.* Typhimurium ΔppGpp expressing RGD was more efficient in suppressing tumor growth than an isogenic strain not expressing RGD (Park et al., 2016). Another study also observed robust colonization when *S.* Typhimurium was engineered for expressing interleukin 15 (IL-15) and two immune checkpoint inhibitors directly into the tumor microenvironment (Augustin et al., 2021). Treatment of mice bearing native mammary gland tumors with *S.* Typhimurium χ455O expressing these immunomodulators significantly reduced tumors and increased survival with minimal toxicity (Augustin et al., 2021).

### 4.4 Delivery of prodrug-converting enzymes

Most chemotherapy drugs are toxic to both tumor and normal tissue; thus, prodrug-converting enzymes have been used in cancer therapy to reduce the adverse effects of toxicity in healthy tissues and maximize the efficiency of chemotherapeutics. These enzymes convert systemically administered prodrugs into their active form to conduct cytotoxic functions. The usefulness of *S.* Typhimurium in delivering prodrug-converting enzymes in the tumor region has been explored (King et al., 2002; Nemunaitis et al., 2003; Friedlos et al., 2008). This strategy delivered *E. coli* cytosine deaminase, which converts the harmless substrate 5-fluorocytosine (5-FC) into the chemotherapeutic agent 5-fluorouracil (5-FU) (Austin and Huber, 1993; King et al., 2002). Co-administration of VNP20009-loading cytosine deaminase (TAPET-CD) and 5-FC efficiently suppressed tumor growth in mice bearing murine colorectal carcinoma and human colon tumor xenografts.

Additionally, bacterial colonization and converting 5-FC to 5-FU by cytosine deaminase were observed in tumor tissue but not healthy tissues (King et al., 2002). However, co-administration of TAPET-CD and 5-FC in three patients having solid or metastatic tumors did not cause tumor growth inhibition. Still, tumor colonization and conversion of 5-FC to 5-FU were observed, indicating that *S.* Typhimurium can produce functional cytosine deaminase (King et al., 2002).

## 5 Combination therapy

The potential of *S.* Typhimurium-mediated immunotherapy and other therapies has been widely explored in the last three decades. Evidence indicates that combination therapy results in more successful treatment than monotherapies, probably due to the multifactorial nature of cancer (Massa et al., 2013; Zhao et al., 2020). *S.* Typhimurium has been extensively explored against cancer, chemotherapy, radiotherapy, and photothermic therapy, among others (Saltzman et al., 1996; King et al., 2002; Bascuas et al., 2018; Miyake et al., 2019; Duo et al., 2023). Previous studies have shown promising results when combining *S.* Typhimurium with chemotherapeutics such as 5-FU, cisplatin, cyclophosphamide, doxorubicin, and vincristine (King et al., 2002; Bascuas et al., 2018; Saltzman et al., 2018; Miyake et al., 2019). Chemotherapy has reduced cytotoxic effects on tumor cells in the G0/G1 phase; however, the population of these cells predominates in established tumors. *S.* Typhimurium A1-R can increase the sensitivity of tumors to chemotherapy, presumably by induction of the cell cycle progression from the G0/G1 phase to the S/G2/M phase. *S.* Typhimurium A1-R was combined with cisplatin and recombinant methioninase in a metastatic osteosarcoma model that does not respond to treatment with cisplatin. Results showed that combining these three elements was more efficient in eradicating metastatic osteosarcoma than individual therapies (Igarashi et al., 2018). Similar results were found when *S.* Typhimurium LVR01 combined with four drugs (cyclophosphamide, doxorubicin, vincristine, and prednisone) significantly inhibited tumor growth compared to LVR01 alone or chemotherapy alone in a murine model of B-cell non-Hodgkin lymphoma (Bascuas et al., 2018). Recently, bioengineered *E. coli* OMVs combining photodynamic/chemo-/immunotherapy improved the antitumor efficacy against triple-negative breast tumors in mice and avoided lung metastasis by co-loading of chlorin e6 and doxorubicin (Li et al., 2023).

Although *S.* Typhimurium and radiotherapy have not been much studied, recent studies have shown an increase in the antitumor activity of *S.* Typhimurium when combined with radiotherapy in mice bearing CT26 tumors (Liu et al., 2016; Gao et al., 2020). *S.* Typhimurium KST0652, a mutant oxytolerant and hyper-attenuated, can express and secret inducer sATF6. This strain was engineered to deliver sATF6 inside tumor cells *via* T3SS in response to the radiation-inducible *recN* promoter. *S.* Typhimurium KST0652 was more effective in tumor targeting, colonizing, and invading than its parental strain. However, KST0652 in monotherapy could not significantly reduce tumor growth (Gao et al., 2020). Exposure to γ radiation significantly decreased tumor growth. Still, complete suppression of tumor growth was observed only when mice inoculated with KST0650 were exposed to γ radiation to induce the expression and secretion of sATF6 (Gao et al., 2020). Primary melanoma tumors were significantly reduced when treated with *Salmonella* as a radiosensitizer delivery vector combined with radiotherapy (Duo et al., 2023). These and other results suggest that the combined treatment of *S.* Typhimurium and radiation produce additional antitumor effects.

Alternatively, combining *S.* Typhimurium with photothermic therapy for treating cancer has also been studied (Chen et al., 2019a; Yi et al., 2020). For example, in a recent study, *S.* Typhimurium YB1 targeting hypoxia combined with photothermal treatment showed greater specificity and efficiency in tumor eradication. The surface of YB1 was coated with nanophotosensitizers (nanoparticles loaded with indocyanine green). Once inoculated into MB49 tumor-bearing mice, this bacterium invaded all tumor tissue. Subsequently, irradiating the tumors with a near-infrared laser directed the elimination of tumors (Chen et al., 2019a). Another study indicated that infrared laser irradiation could kill *S.* Typhimurium-infected tumors. *S.* Typhimurium infection of tumors triggers the infiltration of immune cells, the release of proinflammatory cytokines, and the alteration of the tumor vasculature, leading to thrombosis. In that study, it was found that *S.* Typhimurium-infected tumors had a dark color, which could be used to cause photothermal heating with a near-infrared laser (Yi et al., 2020).

## 6 *Salmonella* outer membrane vesicles in cancer therapy

Bacteria naturally produce and secrete OMVs under physiological and pathological conditions. OMVs of Gram-negative bacteria are spherical two-layer proteoliposomes of diverse nano-sized (10-300 nm) (Beveridge, 1999; MacDonald and Kuehn, 2012; Igarashi et al., 2018) composed of LPS, outer membrane proteins, periplasmic proteins, and phospholipids (Bai et al., 2014). Studies have revealed that OMVs can transport a variety of toxins, metabolites, enzymes, virulence factors, and genetic material (DNA and RNA) (Bai et al., 2014; Batista et al., 2020). The cargo transported by OMVs depends on the growth conditions. For example, A proteomic analysis identified differences in the composition of OMVs of *S.* Typhimurium isolated under two different growth conditions. Among the proteins identified, 14 were specific for OMVs isolated from growth in minimal acidic medium (Bai et al., 2014); suggesting that *S.* Typhimurium modulates the cargo of OMVs to adapt to environmental changes.

Morphologically, OMVs have some similarities to bacterial minicells. Both are anuclear nanometric particles composed of cellular material of their parental cells (Bai et al., 2014; Solomon et al., 2015). Some authors do not discriminate between OMVs and minicells (Cao and Liu, 2020). However, although OMVs and minicells have similarities, they are different. One of the characteristics that differentiate minicells from OMVs is the cell wall. Minicells have a cell wall, while OMVs generally do not. OMVs are naturally secreted during all phases of bacterial growth. At the same time, minicells are derived from aberrant cell division, generated from the inactivation or mutation of genes (mainly the *minCDE* gene) that control the normal process of cell division (Solomon et al., 2015). Furthermore, the minicells are larger than OMVs.

**Figure 4:**
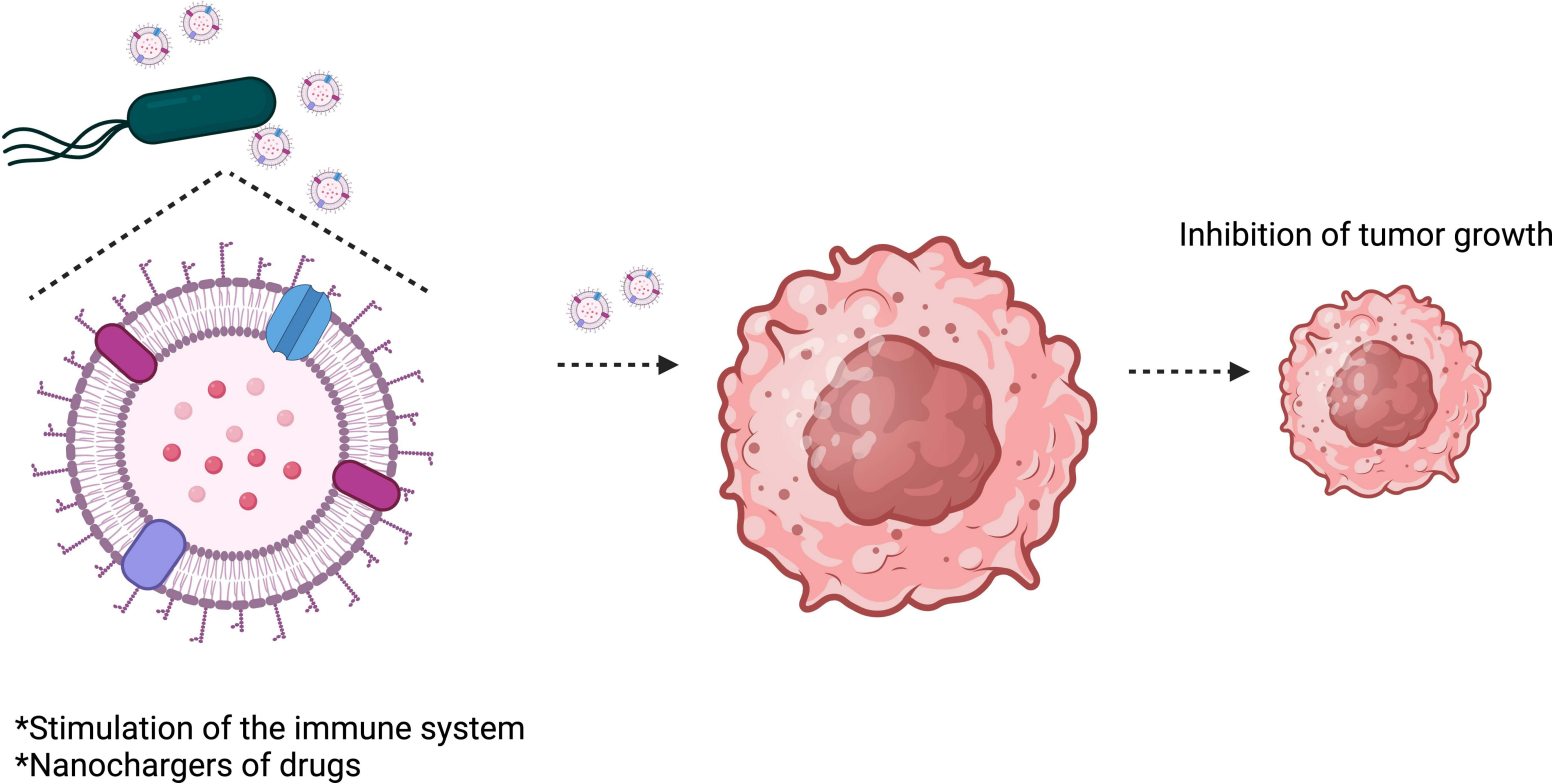
*Salmonella* Typhimurium outer membrane vesicles (OMVs) inhibit tumor growth. OMVs can stimulate the immune system to attack the tumor and can also be used to transport therapeutic drugs to tumors.

OMVs are considered safer than attenuated bacteria and can stimulate the immune system as they comprise most of the immunogens found on the surface of their parent bacteria (Kaparakis-Liaskos and Ferrero, 2015; Kim et al., 2017). They can also act as efficient nanochargers of drugs (**Figure 4**). OMVs have recently emerged as a new strategy for designing treatments and vaccines against diseases, including cancer (Kuerban et al., 2020). OMV-based vaccines have been used in Normandy (Holst et al., 2013) and New Zealand (Sevestre et al., 2017) to control meningococcal serogroup B clonal outbreaks. The data shows that the vaccine has been efficient and safe. However, the use of OMVs in the translational clinic faces some difficulties, such as poor production performance and endotoxic effects.

### 6.1 Production of *Salmonella* OMVs

The large-scale production of OMVs represents a challenge for the use of OMVs for clinical application (Micoli et al., 2023). Under normal conditions, bacteria have a low yield of OMVs for manufacturing vaccines or drugs. The production of OMVs is limited; however, its production can be considerably improved through genetic modifications. For example, OMV production is influenced by protein cargo, membrane proteins and lipoproteins, and LPS composition (Avila-Calderón et al., 2015). All genes related to the bacterial envelope (membrane, peptidoglycan, or LPS) can modulate OMV biogenesis.

In *S. enterica* serovar Typhi, nine genes were reported to modulate OMV secretion. Genes involved with envelope stability (*ompA,nlpI*, *tolR*), LPS biosynthesis (*waaC* and *rfaE*), synthesis and remodeling of the peptidoglycan (*mrcB*), genes involved in cell division (*yibP* and *envC*), stress sensor (*degS*) and the global transcriptional regulator (*hns*) have been associated with OMV biogenesis (Nevermann et al., 2019). Mutations that result in changes in the bacteria surface can result in a hypervesiculated phenotype. *Salmonella* mutants *ΔrfaE*, *ΔtolR*, and *ΔdegS* are associated with enhanced periplasmatic protein secretion. *ΔdegS* mutant, in particular, secrets about 30% more proteins than the wild-type strains. Some mutations, such as *ΔompA*, *ΔtolR*, *ΔdegS*, and *Δhns*, result in bigger OMVs, while *ΔmrcB* results in smaller vesicles, and *ΔwaaC* results in more heterogeneous *Salmonella* OMVs (Nevermann et al., 2019).

It was also described that *S. enterica* serovar Typhimurium mutants lacking *lppAB*, *Pal*, *tolA*, or *tolB* genes increased the production of OMVs (Deatherage et al., 2009). Other authors also observed that the *pagL* gene, coded for Lipid A deacylase, is involved with the intracellular release of OMVs in *S.* Typhimurium (Elhenawy et al., 2016) and several other Gram-negative bacteria (Geurtsen et al., 2005). Understanding the genetic aspects behind OMV biogenesis is the key to determining possible mutation targets to modulate OMV production.

### 6.2 Endotoxic effects of OMVs

The endotoxic effect of OMVs is another challenge for their clinical use. The LPS in OMVs isolated from wild-type strains of *Salmonella* is a fully endotoxic and potent activator of immune-inflammatory responses. The inoculation ip or iv of OMVs isolated from wild-type strains of *Salmonella* can trigger an inflammatory cytokine storm and serious systemic inflammatory responses (Lee et al., 2009; Qing et al., 2020). Therefore, LPS from OMVs must be detoxified for clinical use. Various approaches have been investigated to decrease the endotoxicity of OMVs. Detoxification of OMVs can be reduced by chemical modifications and deletions of genes involved in LPS synthesis. Once isolated, OMVs can be chemically modified to reduce their toxicity. In addition, the endotoxicity of OMVs isolated from *Escherichia coli* BL21 was decreased by their encapsulation in calcium phosphate. These OMVs were less endotoxic but maintained their ability to accumulate in the tumor and their antitumor effects (Qing et al., 2020).

Detoxification of OMVs has been performed by an isolation process based on detergents. The safety of OMV-based vaccines that control meningococcal serogroup B is probably due to LPS detoxification. The isolation of these OMVs is carried out with detergents that help eliminate LPS (Holst et al., 2013); however, the total loss of LPS is not an attractive option for immunotherapeutics against cancer since the complete loss of LPS could considerably reduce the immunogenicity of OMVs and, therefore, reduce their antitumor activity. Thus, genetic modifications in the genes that participate in the synthesis of LPS could solve this difficulty.

It is known that *the msbB S.* Typhimurium mutant has reduced endotoxicity in humans compared with wild strains (Toso et al., 2002). Previously, OMVs isolated from *S.* Typhimurium Δ*msbB* were evaluated for delivery of epitopes of a canine parvovirus protein (Lee et al., 2009; Jiang et al., 2022). OMVs isolated from *E. coli* Δ*msbB* have also been assessed against cancer. In both cases, the isolated OMVs had reduced endotoxicity. Still, they retained sufficient capabilities to stimulate the immune system and were proposed to deliver vaccines or therapeutic molecules (Lee et al., 2009; Kim et al., 2017). Another work reported that OMVs derived from *S*. Typhimurium *ΔppGpp* inoculated by the iv route have low toxicity in mice and antitumor activity. These genetic modifications strongly reduce the endotoxicity of OMVs by less activation of the TLR4 pathway. Furthermore, the antitumor effect and safety of attenuated mutants of *S*. Typhimurium have been widely characterized in different mouse models of cancer (**Table 1**); however, the field of OMVs isolated from these mutants has been little explored. OMVs isolated from attenuated mutants of *S*. Typhimurium could be a promising and safe strategy in cancer immunotherapy.

### 6.3 *Salmonella* OMVs as antitumoral agents

Recent studies have shown that OMVs have potential in cancer immunotherapy and can be used with conventional therapies (Kim et al., 2017; Chen et al., 2020; Aly et al., 2021; Zhuang et al., 2021). OMVs isolated from *S.* Typhimurium 14028 were evaluated as monotherapy and combined therapy with Paclitaxel in an Ehrlich tumor model. Treating mice by i.p route with four doses of 5 µg of OMVs of *S*. Typhimurium 14028 inhibited tumor growth by approximately 90%, while the inhibition with combined treatment was 95%. The observed antitumor effect was accompanied by hepatic degenerative changes, with milder liver damage in mice treated with the combination of OMVs and paclitaxel (Aly et al., 2021). Hepatocyte damage is probably due to the endotoxicity of OMV isolated from the wild-type strain and the toxic effect of the chemotherapeutic drug paclitaxel.

Similarly, CT26 tumor-bearing mice were treated by the i.p route with four doses of 5 ug OMVs derived from wild-type strains of *S*. Typhimurium and *E.* coli. Both OMVs inhibited tumor growth in a dose-dependent manner (Kim et al., 2017). On the other hand, mice bearing CT26 tumors were treated i.v with a single dose of 2.5 μg of OMVs derived from *S.* Typhimurium *ΔppGpp*. Although this treatment significantly inhibited tumor growth, it could not eliminate the tumor. However, treatment of CT26 and 4T1 tumor-bearing mice with a single 2.5 μg dose of *S*. Typhimurium *ΔppGpp*-derived OMVs combined with photothermal therapy resulted in complete tumor clearance after eight days of treatment initiation (Zhuang et al., 2021). These results show the enhanced effect of *S. enterica* OMVs combined with other therapies.

The synergistic therapeutic effect between *S.* Typhimurium OMVs and the chemotherapeutic Tegafur was also explored (Chen et al., 2020). OMVs were initially coated with non-contaminating polyethylene glycol and the RGD peptide, which increases the ability of OMVs to target tumors. Subsequently, these OMVs were coated with polymeric micelles from Tegafur, generating polymeric nanoparticles of OMV. Systemically administered nanoparticles selectively accumulated in the tumor, liver, and kidney. Furthermore, it induced antitumor immunity and significantly inhibited tumor growth in the murine model. The antitumor effects were mediated by activating the antitumor immune response and the release of Tegafur, which induced the death of tumor cells (Kim et al., 2017; Chen et al., 2020). These results suggest that *S.* Typhimurium OMVs and chemotherapeutics can considerably enhance cancer immunotherapy.

OMVs derived from *S.* Typhimurium can also be chemically and genetically modified to load and deliver antitumor drugs to tumor cells. OMVs derived from *S.* Typhimurium and *E*. *coli* are known to contain adhesines recognized by cells and trigger endocytosis (Kim et al., 2017; Nevermann et al., 2019). This inherent advantage of OMVs can be harnessed to deliver antitumor drugs into tumor cells. This approach has been explored with OMV isolates from *E. coli* and *Klebsiella pneumonia* (Gujrati et al., 2019; Kuerban et al., 2020). Recently, OMV was isolated from an *E. coli ΔmsbB* strain expressing the tyrosinase transgene, resulting in melanin-loaded OMVs tested in mice. Combination therapy with 75 ug of melanin-loaded OMVs i.t. and near-infrared light irradiation, eliminated tumors in 4T1 tumor-bearing mice (Gujrati et al., 2019). Using a chemical approach, OMVs derived from *K. pneumonia* were modified to package doxorubicin in human alveolar adenocarcinoma cells (A549 cells). OMVs from *K. pneumoniae* significantly reduced tumor growth (Kuerban et al., 2020). To date, OMVs isolated from *S.* Typhimurium have not been used to load therapeutic molecules. Therefore, it is an exciting approach that warrants investigation.

## 7 Conclusions and future perspectives

The intrinsic characteristics of *S.* Typhimurium make it an ideal strategy for cancer immunotherapy. Preclinical studies have provided strong evidence indicating that *S.* Typhimurium as an anticancer agent is a promising immunotherapeutic strategy. It may cause tumor cell death and induce activation of the antitumor immune response. Furthermore, genetic engineering tools have been made using *S.* Typhimurium as a delivery vector for therapeutic payloads against cancer. Despite the promising results observed in *S.* Typhimurium-mediated immunotherapy, it is likely that due to cancer heterogeneity, combining more than one therapeutic strategy is necessary to achieve successful treatment. Combinatorial approaches to *S.* Typhimurium with other types of anticancer therapy significantly improve the efficacy of cancer treatment. However, clinical trials have yielded unsatisfactory results, indicating that some problems must be solved before *S.* Typhimurium-mediated cancer immunotherapy is applied to humans. One of the remaining problems is that the therapeutic efficacy of *S.* Typhimurium appears closely related to its toxicity; therefore, future efforts must be directed to the careful design of bacterial strains that balance attenuation, toxicity, and therapeutic efficacy.

OMVs in cancer immunotherapy have recently received much attention. Studies have provided strong evidence that OMVs derived from *S.* Typhimurium also have potential in cancer immunotherapy, can be combined with conventional therapies, and are superior to their parent bacteria in terms of safety. Like *S.* Typhimurium, OMVs derived from *S.* Typhimurium can stimulate humoral and cell-mediated immune responses and be used as antitumor agents’ nanocarriers. Promising results have been obtained with OMVs of *S.* Typhimurium in different murine models. However, the clinical use of OMVs faces two major challenges: immunogenicity and low production of OMVs. These problems need special attention.

OMVs derived from attenuated mutants of *S.* Typhimurium could balance immunogenicity and safety. The results show that they could be a highly efficient and safe option. Studying OMVs isolated from well-characterized S. Typhimurium mutants in clinical and preclinical studies could represent a promising strategy. In addition, more in-depth studies are needed on the changes in the tumor microenvironment induced by *S.* Typhimurium and OMVs, the interaction between this bacterium and host immunity, and the use of models that better reflect the diversity and complexity of tumors in patients. A better understanding of these aspects will likely increase the chances of success of *S.* Typhimurium and *S.* Typhimurium-derived OMVs in cancer immunotherapy.

## Author contributions

GPJ: Conceptualization, Data curation, Investigation, Visualization, Writing – original draft, Writing – review & editing. MTPG: Formal analysis, Visualization, Writing – original draft. MB: Conceptualization, Formal analysis, Funding acquisition, Investigation, Resources, Supervision, Validation, Visualization, Writing – original draft, Writing – review & editing.
